# The Book World of Medicine and Science

**Published:** 1894-04-21

**Authors:** 


### The Book World of Medicine and Science.

Books for the Sick: The Therapeutics of Literature.

The virtue that lies in books for the healing of the mind—and of the body through the mind—is well recognised. Certain conditions there are—physical as well as mental—in which the best thing that can be done for the patient is to put in his hands a book which he is likely to read with pleasure. Of course, there are times when it would be unwise to allow reading, just as there are times when opium is contra-indicated. When there is acute pain the solace of literature is of little avail. There are stages of weakness when the intellectual effort of listening—not to speak of reading—is too great. The nurse of experience knows when it would be proper to offer to read aloud to her patient; she can tell when the time has come when the invalid may be permitted to read for himself. And who that has had experience of the soothing as well as of the stimulating qualities of literature will deny to books a place among the therapeutic agents of the world?

But it is not enough to admit that a book may be as useful as a drug. Drugs are classified according to their properties. Their value is tested by observation and experiment, and the results communicated to those who practice the healing art. It is part of the training of the physician to recognise the conditions that call for strychnia, those that call for rhubarb. It is not to suggest that it is possible to define the qualities of the best known books as carefully as the properties of familiar drugs to raise the question whether an attempt to classify books according to the influence which they are likely to exert upon certain conditions of mind and body would not be justified. Take the case of an invalid who has so far recovered that he has just been allowed to beguile the weary hours of convalescence by reading. The invalid asks advice as to what he shall read—by no means an uncommon case. Shall he have recourse to Scott or to Dickens? Will you put in his hands the newest thing in philosophy or the latest sensational novel? No one who has a sense of the subtle forces that underlie literature will give answer without consideration. For the patient for whom Scott would be most suitable Dickens would not be so suitable. In the case of one invalid a "sedative" book is indicated; another invalid is depressed, and needs literature that will stimulate without unduly exciting.

Is it then possible to formulate a system of literary therapeutics—to lay down principles in accord with which a book may be prescribed in the same way that a drugs prescribed? No; it will never be possible to make an exact science of literary therapeutics. Literary products cannot be treated like a plant or a salt. There are essences in books which escape the most careful analysis. Nevertheless, there are one or two broad principles as to the books which are suitable for the sick that may be laid down and applied with considerable confidence. In prescribing a book two factors must always be taken into consideration—the physical condition of the invalid, and his natural and acquired literary tastes. The book for a person recovering from a depressing disease would not be the book for the man on whom a broken limb had entailed a term of rest but whose mental faculties were perfectly healthy. The convalescent schoolboy and the convalescent *savant* cannot be expected to relish and digest the same literary fare.

For the rest, one must rely upon a judgment informed by a knowledge of the best literature. For this reason, were it for no other, it is desirable that those who tend the sick should have some knowledge of books. It is easy to realise how in this matter ignorance may be the occasion of harm, even of serious harm. For example, to permit an invalid whose nerve force has been seriously undermined to indulge in a course of highly sensational novels might retard recovery very seriously. To permit a sick student to resume intellectual work until the physical energy which is the condition of intellectual energy has been restored might prove the source of lasting injury to mind and body.

It is then of real importance in the case of invalids that the books they read should do them good and not harm. Why should not the literary food of those toward whose recovery to health our efforts are directed not be the subject of as much consideration as their material food? Every day it is being brought more clearly to light that health of body and health of mind are intimately associated; and few things have a more vital relation to the *mens sana* than the books we read.

### BOOK REVIEWS.

An Indispensable Book.

"Legal Hand-book for the Use of Hospital Authorities." L. S. Bristowe, M.A., Oxon., Barrister-at-Law, author of "A Treatise on the Mortmain and Charitable Uses Act, 1891," and joint author of "The Law of Charities and Mortmain" (Tudor's "Charitable Trusts"). (London: Reeves and Turner, 1894.)

All managers of hospitals will welcome Mr. Bristowe's carefully-arranged and equally carefully written work. In its modest preface the author apologises for its appearance; but, in our opinion, no apology whatever is needed, for, after a careful perusal of it, we are altogether at a loss to discover any legal difficulty which can possibly present itself to those in charge of our hospitals which is not fully and accurately dealt with in its pages. The laws at present in force which regulate the constitution and incorporation of hospitals are tersely, yet lucidly, set forth and explained—not in legal jargon, but—in words "to be understanded of the people." Mr. Bristowe has also spared no pains in his treatment of the various enactments which bear upon the creation and management of hospital funds; while governors, members of committees, and hospital officials generally will find the law relating to their duties and liabilities carefully stated and explained. Every day, in all ranks and professions, books of reference and text books of approved merit are essential to the efficiency of everybody's daily work. Like Burdett's "Hospital and Charities Annual," this book should find a place in the board-room and library of every hospital and institution, as well as on the shelves of public and private libraries throughout the British Empire. No one who studies it will ever willingly be without this book again, and had it been published ten or fifteen years ago it must have saved many managers much expense and trouble. On reading it carefully through the trained and capable secretary will wonder how he has managed to get on without such a book and will feel immensely indebted to the author for publishing it. The "Hospital Legal Handbook" is surprisingly accurate and complete, almost an education in itself, and contains a mine of useful information obtainable nowhere else in so handy a form. An admirable index brings to a close a work which we can confidently recommend to those for whose use it has been written.

Epidemics, Plagues, and Fevers: Their Causes and Prevention. By the Hon. Rollo Russell. (London: Edward Stanford, 26 and 27, Cockspur Street, Charing Cross, S.W. Price, 16s. net.)

The Hon. Rollo Russell's book on epidemics, &c., pretends to no new lights on the subject. Nevertheless, this is a most convenient and useful volume, in that it gives in a convenient and compact form the opinions and experiences of the most approved specialists on the various diseases it deals with. As it is not too technical in phraseology, it is suitable for the ordinary householder as well as the student of infectious diseases, and to both will prove a convenient guide on sanitary questions.

